# Supplementary material for: Acceptance and compliance with micronutrient powder (MNP) among children aged 6–23 months in northern Nigeria
Source: PLOS Glob Public Health. 2022 Oct 17;2(10):e0000961. doi: 10.1371/journal.pgph.0000961 (PMC10022258; doi:10.1371/journal.pgph.0000961)
Supplement: S1 Dataset — (ZIP) [file pgph.0000961.s008.zip › S8_Dataset/Direct Observations.zip!/Direct Observations/270615_K_DW_SA_1_DO.pdf]

1.1.1 Instrument F –Direct Observation Form

<<Instrument F Direct Observation Form>>
**PI Name:** PragyaMathema,Stephen Kodish<br>**Study Title:** Formative research to develop an infant and young child feeding (IYCF) intervention with micronutrient powder (MNP) for children aged 6 – 23 months<br>**PI Version/Date:** Version1.0/January 24, 2015

Date: 27/06/15Location: Kango

Data Collector: Samira Adamu

Main goals of this direct observation are to answer the following questions:<br><br>1. In <u>this</u> household, what would be the best way(s) to give nutritional supplements to <u>young children</u>? Why?<br><br>2. In <u>this</u> household, describe the food sharing that you observe throughout the meal. Are there any <u>predictable</u> patterns?<br><br>3. In <u>this</u> household, who is favored and how is this favoritism expressed during meal preparation and eating?

Household Composition Table (Fill out <u>as</u> you do data collection)

INITIALS	SEX (M/F)	AGE (estimated)	Relationship to household	Description (not necessary)
BB1	F	8months	Youngest child	Baby girl wearing green pants
BB2	F	8months	Youngest child (twin sister to the youngest child)	Baby girl wearing orange pant
M1	F	24years	1st wife	Wearing green and red attire
M2	F	20years	Mother of child and second wife	Wearing orange top with blue wrapper
BG	F	3years	3rd child (step sister)	Wearing blue top and pink skirt
BG2	F	7years	Cousin	Plump and wearing light green attire
YL	F	14years	Niece to the 1st wife	Wearing yellow veil and coffee brown wrapper
F	M	32years	Husband	Wearing dark green kaftan

B1	M	5years	Step brother	Wearing peach coloured shirt

PARTICIPANT ID: _________

TIME	ACTOR INITIALS	RECIPIENT INITIALS	WHAT HAPPENED	FOOD	Reactivity
8:10	M1		Processing sorghum to remove shaft using a stainless bowl without water		
	M	BB2	Carrying the child on her laps sitting on the stool		
	BB1		Playing with a small stainless plate on the floor		
8:15	M1		Still Processing sorghum to remove shaft using a stainless bowl without water		
	M	BB2	Sitting on a stool and breastfeeding the child	Breast milk	
	BB1		Playing near a pot of maize soaked with hot water		
	M1	BB1	Carrying the pot away from the baby		
8:20	BB2		Crawling around the house on the floor		
	BB1		Playing with sieve and small stainless plate on the floor		
	M1		Still Processing sorghum to remove shaft using a stainless bowl without water		
	M		Pouring hot water into a bathing basin		

TIME	ACTOR INITIALS	RECIPIENT INITIALS	WHAT HAPPENED	FOOD	Reactivity
8:25	M1		Still Processing sorghum to remove shaft using a stainless bowl without water		
	BB2		Crawling around the house		
	M		Adding cold water to the hot water in the bathing basin		
	BB1		Playing with sieve and shaft of sorghum processed by the step mother		
8:30	M	BB1	Bathing the child		
	BB2		Playing with the water in the bathing basin		
	M1		Adding cold water to the processed sorghum		
	BB1		Sitting in th bathing basin with water after bath		
	BB2		Still playing with the water		
	M		Bringing a basket of baby's cosmetics to make up the child		
	M1		Washing and decanting the water to remove excess shaft along with the water		
8:35	BB1		Still in the bathing water		
	BB2		Still playing with the bathing water while sitting on the floor		
	M		Removing clothes from the baby's basket of cloth		
	M1		Sieving and draining the excess water and shaft from the sorghum		
	M		Removing clothes from baby's basket of clothes		
8:40	M	BB1&BB2	Carrying the child on her laps and putting the other child in the bathing water		
	M1		Sweeping her room while her child came in crying		
8:45	M		Sitting on a stool, breast feeding	Breast	

TIME	ACTOR INITIALS	RECIPIENT INITIALS	WHAT HAPPENED	FOOD	Reactivity
			then child and rubbing Vaseline on her body	milk	
	BB2		Sitting in the water bah basin and watching the mother		
	M1		Packing dirty clothes from the ground		
8:50	M	BB1	Breastfeeding the child, sitting on a stool and rubbing powder on her face and neck	Breast milk	
	BB2		Still sitting in the water bath basin and watching them		
	BG		Clapping hands and smiling at the child		
8:55	M	BB1	Wearing clothes for the child and still breast feeding the child	Breast milk	
	BB2		Still sitting in the water bath basin and watching them		
	M1		Packing dirty plates and bowls into a big basin		
9:00	M	BB1&BB2	Kept the child on the floor and started bathing the other child		
	M1		Washing the dirty plates packed in the basin		
	BG2		Playing with the children		
9:05	M	BB2	Bathing the child		
	M1		Washing then dirty plates in a basin and putting them in a basket to drain		
	BB1		Sitting on a plastic bowl and stooling		
	BG2		Singing and playing with then children		
9:10	M	BB2	Carrying then child on her laps and rubbing Vaseline		
	BB1		Sitting on a plastic bowl and stooling		
	M1		Still Washing then dirty plates in a basin and putting them in a basket to drain		

TIME	ACTOR INITIALS	RECIPIENT INITIALS	WHAT HAPPENED	FOOD	Reactivity
	BG2		Singing and cutting sticks on the floor for the children as they are all smiling		
9:!5	M	BB1	Holding water in a kettle and carrying the child to a corner of the house to clean her up		
	BB2		Still sitting on a plastic bowl and stooling		
	M1		Washing, rinsing and draining plates in a basket		
9:20	BG		Singing and playing with the twins		
	M1		Rinsing and draining stainless bowls		
	M	BB1	Breastfeeding then child while sitting on a stool	Breast milk	
	BB2		Sitting on the plastic bowl and stooling and also playing with a plastic hand fan		
9:25	M1	M	Sitting the child on the floor and giving her a plastic hand fan		
	BB2		Still sitting on the plastic bowl and playing		
	M1	M	Giving her plates and bowls		
9:30	M	BB2	Holding a kettle of water and carrying the child and the plastic stooling bowl to a corner of the house		
	M1		Carrying a basket of washed plates and bowls to her room		
	BB1		Crawling around in front of the mother's room		
	BG	BB1	Playing with the child and smiling		
9:35	M	BB2	Wearing clothes for the child		
	BB1		Crawling around in front of the mother's room		
	M1		Pouring waste water on the floor		

TIME	ACTOR INITIALS	RECIPIENT INITIALS	WHAT HAPPENED	FOOD	Reactivity
	BG2	BB2	Carrying the child and singing while going out of the house		
	BG1		Entering the house crying and shouting the mother's name		
	BB1		Crawling and playing with a wrap of stick		
9:40	BB1		Crawling and playing with the sister		
	BG1		Playing with the child		
	M		washing her child's clothes in front of her room		
	M1		Lying on a mat in front of her room		
9:45	BB1&BG		Crawling and playing together on the floor		
	M		Lying on a mat and gisting with her rival		
	M1		Standing and drying her clothes while gisting with her rival		
9:50	BB1		Playing alone with rubber hand fan and crawling		
	M		Lying on a mat in front of her room		
	M1		Leaving the house		
9:55	BB1		Playing with the direct observation form		Playing with direct observation form and pen
	M	BB1	Carrying the child away from me		
10:00	BG		Entering the house and playing with the child and smiling		
	BB2		Happily smiling with the cousin		
	M		Giving a young girl a stainless basin with half full of sorghum to take to her mother		
10:05	BG	BB1	Standing the child on her lap and singing for her		

TIME	ACTOR INITIALS	RECIPIENT INITIALS	WHAT HAPPENED	FOOD	Reactivity
	M		Lying on a mat in front of her room		
10:10	M1		Entering the house and talking with her child		
	M		Lying on a mat in front of her room		
	BG	BB1	Playing with the child and smiling		
10:15	M1	BG	Instructing her to collect waina pan from the neighborhood		
	M		Lying on a mat in front of her room while the child is playing with the scarf		
10:20	M		Sitting on a stool in front of the house and gisting with her rival who is lying down on a mat		
	M1	BB1	Breastfeeding the child while lying on the mat	Breast milk	
10:25	M1	BB1	Still breast feeding the child still lying on the mat	Breast milk	
	YL	BB2	Entering and carrying the child, giving the child to her mother and I was told she niece to the first wife		
	M		Still sitting on a stool in front of her room		
10:30	M1	BB1&BB2	Breastfeeding the children together at once while lying down	Breast milk	
	BB2&BB1		Suckling the mother's breast and playing with each other's hand	Breast milk	
10:35	BB1		Crawled towards me and dragged my folder		Dragging my folder
	YL		Carrying the child from me		
	M	BB2	Breastfeeding the baby and lying on the mat with the baby		
	M1		Telling the lady to bring the child to her place		
10:40	M	BB2	Lying on the mat and breastfeeding the twin child while the child is sleeping and sucking	Breast milk	

TIME	ACTOR INITIALS	RECIPIENT INITIALS	WHAT HAPPENED	FOOD	Reactivity
	M1	BB1	Carrying the child on her lap and singing		
10:45	M1	BB1	Still playing with the child and she is smiling happily		
	M&BB2		Both are sleeping on a mat in front of the room		
10:50	M1		Sleeping on the mat		
	BB2		Sleeping on the mat with their mother		
	M1	Bb1	Carrying the child to a neighborhood		
10:55	M1	BB1	Holding the child and gisting with the neighbors while collecting a tray from them		
11:00	M1	B1	backing the baby and carrying a kettle of water		
	M		Sleeping on a mat in front of her room		
	BB2		Sleeping beside the mother		
11:05	M1	BB1	Backing the baby and rinsing a pant while the baby is sleeping		
	M		Sleeping on a mat in front of her room		
	BB2		Sleeping alongside mother		
11:10	M1	BB2	Backing the child and sitting on the stool while the baby was sleeping		
	M&BB2		Both are still sleeping on the mat		
11:15	M&BB2		Both are still sleeping on the mat		
	M1	BB1	Backing the baby and sitting on a stool		
11:20	M1		Backing the baby and carrying the child to the neighborhood while the baby is sleeping		
	M&BB2		Both are still sleeping on the ma		
11:25	M1		Backing the baby in the		

			neighborhood and sending a child to buy her kanwa (potash)		
11:30	M1		Backing the baby who is still sleeping while standing in the neighborhood		
11:35	M&BB2		Both are still sleeping on the mat in front of her room		
	M1	BB1	Backing the baby and sitting on the stool in front of her room		
11:40	M1	BB1	Backing the baby and sitting on the stool in front of her room		
	M1&BB2		Both are still sleeping on the mat in front of her room		
11:45	M1	BB1	Backing the child and standing at the entrance of the house and gisting with an aged woman<br>The aged woman is the wife of the father-in-law		
	M1		Sitting on the mat in front of her room		
	BB2		Still sleeping on the mat beside the mother		
11:50	M	BB2	Carrying the child on her shoulders and folding the mat		
	M1	BB1	Sweeping the compound of the house and backing the baby who is still sleeping		
	BG		Playing in front of the mother's room with her scarf		
11:55	M1	BB1	Backing the child and still sweeping the compound		
12:00	M1	BB1	Backing the child and sitting on a stool		
12:05	M1		Lying a child on a mat in front of the rival's house		
	M		Sleeping beside the children and sleeping		
12:10	BB1		Sleeping on the mat		
12:15	BB1&M		Still sitting on the mat while the mother sits beside her		
12:20	BB1&M		Still sitting on the mat while the mother sits beside her		

12:25	BB&M		Sleeping on the mat while the mother sleeps beside her		
12:30	M1		Lying on a mat in front of her room		
12:35	BB&M		Sleeping on the mat while the mother sleeps beside her		
	M1		Lying on th mat in front of her room		
12:40	F		Carrying a hoe from the farm, greeted me and entered her room		
	BB		The child is still sleeping on the mat		
	M		Putting water into a basin for the husband to bath		
12:45	M	BB1	Carrying the child on her laps, sitting on the mat while the child is just looking at the mother and touching her mouth		
12:50	M	BB1	Breastfeeding the child while sitting on the mat	Breast milk	
	BG		Pouring a bucket of water into water storage drum that has a cover		
12:55	M1		Setting of fire, arranging three stones		
	M	BB1	Sitting in the room and breast feeding the child	Breast milk	
1:00	BB1		Sitting on the mat and playing with hand fan		
	M1		Setting the fire		
1:05	M		Mixing maize flour paste which was made in the early hours of today adding salt and yeast to the paste		
1:10	BG&B1	BB1	Playing wither siblings with broom sticks		
	M1		Adding half spoon of groundnut oil to each of the pan		
	M		Still mixing maize flour paste with salt and yeast		
	M1		Putting full of the properly mixed flour paste to each of the frying pan		

			and allowing it to fry on one side while turning the other		
1:15	M	BB1	Breastfeeding the child while standing	Breast milk	
	M1		turning the waina to the other side for it to fry well		
	M1		Removing the ones that have fried and putting It in a bowl with cover, and adding groundnut oil to the empty well and immediately adding of paste		
1:20	M	BB1	Sitting on a stool and breast feeding the child	Breast milk	
	M1		Turning the waina upside down to fry very well and removing the properly fried ones and adding oil to the empty well and immediately adding more paste		
1:25	M		Sweeping her room		
	M1		Turning the waina upside down to fry very well and removing the properly fried ones and adding oil to the empty well and immediately adding more paste		
1:30	M	BB1	Breastfeeding the child while sitting on the stool	Breast milk	
	M1		Still taking the same process of frying waina		
	BG	BB1	Playing with the child while she suckles	Breast milk	
1:35	BG	BB1	Still playing with child		
	BB1		Playing with her sister and stooling on the mat, the mother carried her and keeps her on a stooling rubber bowl but she is crying		
1:40	BG		Eating waina with grounded pepper		
	M	BB1	Washing the child with water In one corner of the house still taking the same process of frying waina		
	M1		Still taking the same process of		

			frying waina		
	YL		Sweeping aunt's room which was messed up by the children		
1:45	BG	BB1	Feeding the child with waina and pepper as she also eats	Wainan maize	
	M1		Still taking the same process of frying waina		
	M		Carrying a bucket of water to the toilet		
1:50	BG	BB1	Still feeding the child waina and pepper while the child sits very close to the step mother's leg		
	M1		Still taking the same process of frying waina		
	BG2	BB2	Carrying the twin child and bringing her to the house from the neighborhood		
	BB1		Crying as she is been carried away from the step mom by the cousin sister		
	BB2		Sitting on a stooling rubber pan and crying		
1:55	YL		Taking the same process of frying waina		
	BG	BB	Jumping and clapping hands for the child in the room		
2:00	M	BB2	Carrying the child and giving her water		
	YL		Still taking the same process of frying waina		
2:05	B	BB1	Carrying the child to her twin sister		
	BB2&BB1		Crying and crawling to their mother		
2:10	BB1&BB2		Still crying and crawling around the mother while she is praying		
2:15	BB1&BB2		Still crying while the mother is praying in her room		
2:20	M	BB1&BB2	Feeding both children on separate breast at the same time	Breast milk	
2:25	M	BB1	Sitting on the mat and feeding the child	Breast milk	
	M1		Still taking the same process of		

			frying waina		
	BB2		Playing with the rubber hand fan		
2:30	M		Dissolving half tablet of flagyl and dissolving it in the water, carried the child on her laps and gave her the medicine		
	BB1		Crawling towards me and trying to touch my pen		
	M1		Still taking the process of frying waina		
2:35	BB1		Crying on the mat, mother carried her and was feeding her	Breast milk	
	M1		Still taking the same process of frying waina		
2:40	BB1&BB2		Crawling around in the house		
			Sitting in front of the step mother's corridor		
2:45	BB1		Sitting on the floor and watching her mother fry waina		
	M		Still taking the process of frying waina		
2:50	BB1		Playing with an empty bucket		
	M		Still taking the process of frying waina		
2:55	M1	BB1	Carrying the child on her laps and singing for her		
	BG	BB2	Holding hands and playing with the child		
	YL		Counting the waina and taking it for hawking		
	M		Putting the remaining waina in a bowl and covering it for family consumption		
3:00	BG	BB1	Eating waina with the child	Waina maize	
3:05	BB1		Eating waina		
3:10	M	BB1	Feeding her with waina	Waina maize	
	M	BB1	Sitting on the mat and crying, the	Breast	

3:15			mother carries her and is breastfeeding her	milk	
3:20	M	BB1&BB2	Feeding the child with waina	Waina maize	
	M1		Is packing the used bowls and spoons after frying waina		
	BB2		Twin child is stooling on the floor while the child is eating waina		
3:25	M		Washing the used dirty plates		
	BB1&BB2		Playing together with one rubber hand fan		
3:30	BB1&BB2		All playing on the mat together		
3:35	BB1&BB2		Crawling around the house and playing with buckets, slippers and my hand bag, but the step mother carried them away		Touching my hand bag
3:40	M	BB1&BB2	Carrying them and sitting on the mat		
3:45	M	BB1&BB2	Giving the twin child water	Water	
3:50	BB1		Crawling and crying, the mother carrying her and feeding her while standing	Breast milk	
	M1		Trading with a woman, giving her sorghum while she offered her baby clothing in return		
3:55	M		Carrying the twin child and giving her water while the step mother carries the child	water	
4:00	M1	BB1&BB2	Bathing the children with boiled herbal plant water; I was told it's because the twin child has diarrhea		
4:05	M1	BB1	Feeding the child while the twin child having diarrhea remains in the water bath basin		
4:10	BB1		The child is sleeping on the mat		
4:15	BB1&BB2		Both sleeping on the mat together		
			The mother holding a kettle with water and performing ablution		

4:20	BB1&BB2		Both children sleeping on the mat whereas both mothers/wives are at the neighborhood		
4:25	BB1&BB2		Children sleeping on the mat while wives are at the neighborhood		
4:30	BB1&BB2		Children are still sleeping		
4:35	BB1&BB2		Children are still sleeping		
4:40	BB1&BB2		Children are still sleeping		
4:45	BB1&BB2		Children are still sleeping		
4:50	BB1&BB2		Children are still sleeping		
4:55	BB1		Still sleeping with twin sister on the mat		
	M1		Sweeping and arranging the kitchen		
	M		Still at the neighborhood		
5:00	BB1		Still sleeping with twin sister on the mat		
5:05	BB1		Still sleeping with twin sister on the mat		
5:10	BB1		Still sleeping with twin sister on the mat		
5:15	BB1		The child is sitting on the mat alongside the twin sister sitting on the mat also		
5:20	M1		Setting the fire		
	M	BB2	Breastfeeding the twin child	Breast milk	
	BB1		Sitting on the mat and playing with sand		
5:25	M1		Putting water in a pot and placing it on fire		
	BB1		Sitting on the mat and playing with sand		
	M1	BB1	Giving the child water and backing		

5:30			her		
	M	BB2	Backing the twin child and arranging the house		
5:35	M1		Backing the twin and washing soaked maize in hot water which has been soaked since morning Draining it in a calabash so that it can dry and be used for waina for the next day		
5:40	M1		Backing the child and adding firewood to the fire		
5:45	M1		Making a paste from grounded sorghum flour by adding water and mixing it properly to obtain a semi solid mixture		
	M	BB1	Carrying the twin child on her lap and breastfeeding her	Breast milk	
5:50	M	BB1&BB2	Breastfeeding the child and twin sister on th mat	Breast milk	
5:55	M	BB1	Breastfeeding the child on the mat while the twin sister lays on the mat	Breast milk	
6:00	M	BB1	Still breast feeding the child while the twin sister eats dankaule and the child refuses to eat	Moringa	

NOTES

I asked if she has administered the MNP throughout the day because I have not seen her doing it

<u>Response (1 st wife)</u>

"Yes *she has taken it and we prefer giving her in the morning when the kunu is very fresh and when it reaches afternoon, it gets fermented".*

<u>Response (2nd wife)</u>
"I usually give the kunu dawa in the morning, so I use the opportunity to administer the MNP into a small portion of the kunu, after mixing, I give the specified child. When she finishes, I continue giving them the kunu together and I also did today".

I also asked her how she administers the MNP

"*I prepare kunu in the morning and when it is too thick that the children won't like it, I add a little water but that does not make it watery because the twins don't like watery kunu. I fetch small portion of kunu, open the MNP and pour all to the fetched portion mix properly and give her to finish it all. But she has been sick for like 3-5days ever since then. I have to force to finish it and in the process some pour on her body. They don't like kunu these days because the twin is sick and not well these days".*

Primary Questions (Complete <u>after</u> you complete data collection):

From what you have seen observing THIS HOUSEHOLD; please answer the following questions in detail:

Question	Write your detailed response here
1. In this household, what would be the best way(s) to give nutritional supplements such as an MNP to young children to ensure compliance?<br><br>Why?	Through the 2 wives most especially the first wife<br><br>Because both of them collectively look after the children and it seems the first wife is more attentive to the children and takes most of the responsibilities in the house. The y also share mutual respect between them because the 2nd wife (mother of child) really respects and take the word of the 1st wife serious
2. In this household, who is favored during meal preparation or feeding?<br><br>How is this favoritism expressed?	There was no favoritism to my own understanding based on my observation because the children are just practicing to eat. And I was not opportune to see the feeding pattern since it is fasting period
3. How could we promote the MNP supplement for this family that would attract their attention?<br><br>Who would they listen to?	Through the health care providers because it seems they really care about the health of their children based on my observation , the twin child having diarrhea was not just left at home but taken to the hospital and given drugs alongside 2injections. And I was given a warm welcome because I said I was from FMOH<br><br>The wives will listen to the husband and the husband is exposed to some level of civilization, he has a good understanding

4. In this household, what would be the best ways to promote the MNP but limit how much it would be shared?<br><br>Why?	I think since the other caregiver doesn't have a child of 6-23months, maybe she should be given MNP for one of the twins while the mother holds for the other.<br><br>Because the first wife is very attentive and likes carrying the children and the children likes her too, I feel she will help in proper administration to one of the twins and limit its sharing among the twins. She will also make sure that older siblings don't temper with it because I have seen/observed how she keeps pepper, oil and magi out of the reach of the children
5. What other issues and concerns did you see with this family that are important for us to consider with regard to introduction of an MNP?<br><br>Why?	The major concern I see and observed in this family is that the children of the required age are twins and both of them are always demanding the same thing together at almost the same time<br><br>Because if proper sensitization and awareness in the usage of the MNP is not done properly, there is a tendency of MNP sharing since they are together, she may decide to mix the MNP for the children in the same place when the product has been scaled up to ease her work.
6. Based on your experience observing this child today, do you believe reactivity negatively impacted your ability to see the food-related behaviors of the child?<br><br>Please explain your answer in detail.	No it has not hindered me from observing the actual behavior of the child towards food<br><br>Because the child is just 7 months old and mostly feed on breast milk and a little of other food like waina
